# Spectosis: One small step for NLRP3 but a giant leap for human mature red blood cells

**DOI:** 10.1002/ctm2.70457

**Published:** 2025-08-17

**Authors:** Yaozhen Chen, Jing Zhang, Xingbin Hu

**Affiliations:** ^1^ Department of Transfusion Medicine Xijing Hospital, Fourth Military Medical University Xi'an China

1

Human mature red blood cells (RBCs) play a crucial role in oxygen delivery. These biconcave disk‐shaped cells, supported by cytoskeletal components, must maintain their unique morphology to facilitate gas exchange.^1^ When the cytoskeletal structure is compromised by mechanical, chemical, genetic, immunological, or other factors, the release of haemoglobin, heme, or cellular remnants from RBCs poses a significant threat to the body, leading to severe anaemia, fever, inflammation, and acute renal and liver dysfunction.^2^ In intravascular haemolysis, the complement cascade is traditionally understood to regulate this process. Serine protease cascades converge to produce C3a, C5a, C3b/iC3b and other components that assemble the membrane attack complex (MAC), which forms pores on the membranes of RBCs.^3^ However, the events following the pore formation by MAC have been largely ignored. In the recent study, we demonstrate that the activated complement mediates an intracellular miniNLRP3‐ASC‐caspase‐8 assembly in human mature RBCs, which leads to the proteolysis of β‐spectrin and the breakdown of cytoskeletal structures.^4^

NLRP3 was characterised over 20 years ago, and significant progress has been made by scholars in this intriguing field.^5^ However, nearly all previously reported data pertain to nucleated cells. In these nucleated cells, the NLRP3‐ASC‐caspase‐1 complex promotes the maturation of interleukin (IL)‐1β and IL‐18, as well as facilitating the proteolysis of gasdermins (GSDMs) for pyroptotic pore formation on the membrane.^5^ Our observations in mature RBCs indicate that miniNLRP3‐ASC complex recruits caspase‐8 to degrade β‐spectrin, suggesting the existence of additional non‐canonical targets located downstream of the NLRP3‐ASC complex. In addition, it's well known that ATP, lipopolysaccharides (LPS), nigericin and other stimuli can activate NLRP3 to regulate inflammation response.^5^ Here, we found that ABO blood group reactions through the complement cascade also activate NLRP3. Thus, these data suggest the NLRP3 activation models are more interesting and complex than assumed. There are several homologs of NOD‐like receptors (NLRs) in nucleated cells besides NLRP3, and it remains unclear whether they are involved in the biology of RBCs, as NLRP12 was excluded in the present study.

Human mature RBCs possess a unique cellular structure, lacking nuclei and organelles such as mitochondria, endoplasmic reticulum, and lysosomes, which distinguishes them from nucleated cells. These characteristics suggest that the death of mature RBCs may not conform to the same patterns observed in nucleated cells. Programmed cell death (PCD) has been studied for over a century, with various forms characterised for nucleated cells, including apoptosis, necrosis and pyroptosis.^6^ For mature RBCs, a Ca^2+^‐dependent PCD called eryptosis has been proposed as an apoptosis‐like death mechanism during the degradation of senescent RBCs.^7^ Additionally, pore‐forming toxins induce a necroptosis‐like PCD in mature RBCs.^8^ Nevertheless, more details in the PCD of RBCs are far from fully understood. Recently, we reported that the miniNLRP3‐ASC‐caspase‐8 signalling axis played a pivotal role in regulating PCD in mature RBCs through the cleavage of β‐spectrin (Figure 1). Although we characterised the process of spectosis in RBCs during haemolysis, other forms of PCD have not been thoroughly examined. Given the emergence of several novel, defined modes of cell death in recent years, it would be intriguing to investigate them in mature RBCs.

**FIGURE 1:**
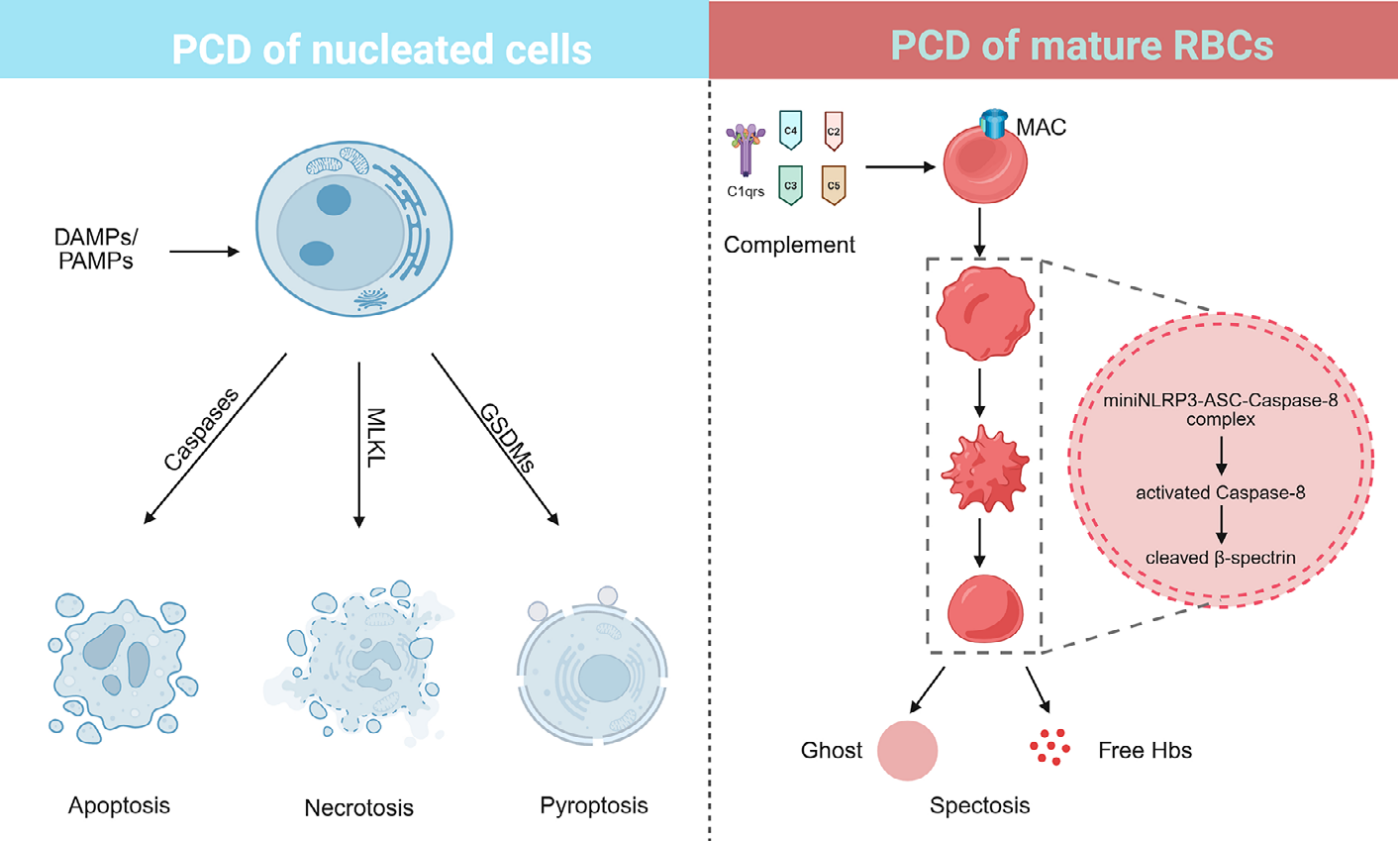
Schematic drawing of human mature RBCs spectosis and programmed death in nucleated cells. Various forms of PCD, such as apoptosis, necrosis, and pyroptosis, are characterised in nucleated cells. Damage‐associated molecular patterns (DAMPs) and pathogen‐associated MPs (PAMPs) stimulate these nucleated cells, leading to the activation of caspases, mixed lineage kinase domain‐like protein and gasdermin. In contrast, in human mature (RBCs, the activation of complement forms MAC and activates intracellular miniNLRP3, resulting in the assembly of apoptosis‐associated speck‐like protein and caspase‐8. Furthermore, active caspase‐8 hydrolyses β‐spectrin, compromising the cytoskeleton and inducing the release of haemoglobin and the formation of cellular ghosts. This type of PCD is termed spectosis in human mature RBCs during complement‐mediated haemolysis.

Hemolysis refers to the destruction of mature RBCs under various stressors. In addition to haemolysis mediated by the complement cascade, mechanical processes, such as extracorporeal membrane oxygenation or extracorporeal circulation in clinical settings, can also damage RBCs. Chemical agents, including phenylhydrazine and ether, are known to induce haemolysis. Furthermore, infections caused by pathogens, including viruses, bacteria or malaria, can lead to both haemolysis and anaemia. Despite the diverse etiologies, RBCs typically exhibit a loss of membrane skeletal integrity prior to damage. A comprehensive understanding of common mechanisms of membrane destruction and repair will undoubtedly enhance therapies for haemolysis and anaemia. Genetic disorders affecting mature RBCs should not be overlooked; for instance, a pedigree of hereditary spherocytosis was analysed in this report, although the mutation and damage sites differ from those observed in complement‐mediated haemolysis. In addition, conditions such as sickle cell anaemia and thalassemia involve membrane injury in mature RBCs; however, it remains unclear whether the miniNLRP3‐ASC‐caspase‐8‐β‐spectrin axis is involved in them.

Since complement‐cascade reactions are primary contributors to intravascular haemolysis, targeting the complement system (specifically C3, C5 or factor B) has been increasingly explored in recent years.^9^ However, these extracellular inhibition strategies do not always yield successful outcomes in the clinical applications. In the present study, we observed that the blockade of NLRP3 or caspase‐8 can mitigate complement‐mediated haemolysis in a mouse model of acute haemolytic transfusion reaction (AHTR). Furthermore, the combined inhibition of both intracellular and extracellular signalling pathways proves to be more effective than the blockade of a single pathway in preventing AHTR. Thus, these data suggest that targeting intracellular signalling pathways may represent a viable option for treating haemolytic diseases. Additionally, we discovered that spectosis signalling is implicated in autoimmune haemolytic anaemias (AIHA) and paroxysmal nocturnal haemoglobinuria (PNH). Based on these findings, we propose a novel target and strategy for the therapy of haemolytic diseases in the future, particularly in complement‐positive AHTR, AIHA, and PNH.

Recent studies have unveiled the dynamics of cellular membrane damage and repair in nucleated cells, which hold significant implications for therapy.^10^ If these dynamics can be extended to human mature RBCs, their remarkable deformability makes this extension even more critical. The balance between membrane damage and repair in mature RBCs warrants in‐depth investigation. At the final stage of haemolysis, damaged RBCs transform into ghosts.  In addition to heme metabolism, the mechanisms involved in clearing these ghosts will determine the overall outcome. Undoubtedly, macrophages and CD47, along with other ‘don't eat me’ signals, will play a critical role in the clearance of ghosts during haemolysis; however, more detailed information is waiting for further elucidation.

## AUTHOR CONTRIBUTIONS

Xingbin Hu and Yaozhen Chen conceived and wrote the manuscript. All authors contributed to the additional writing and editing of the manuscript.

## CONFLICT OF INTEREST STATEMENT

The authors declare no conflict of interest.

## ETHICS STATEMENT

The current manuscript did not involve animal experiments or human clinical trials and was not unethical.
